# Dysphagia Lusoria: A Rare Cause of Adult Dysphagia

**DOI:** 10.7759/cureus.28648

**Published:** 2022-08-31

**Authors:** Venkata Jaya Divya Tenneti, Anoop T, Arun Reddy

**Affiliations:** 1 Department of Vascular Surgery, Manipal Hospitals, Bengaluru, IND; 2 Department of Cardiothoracic Surgery, Manipal Hospitals, Bengaluru, IND

**Keywords:** young adult, surgical management, ct chest angiography, vascular compression, dysphagia

## Abstract

Dysphagia lusoria is an unusual condition characterized by difficulty swallowing secondary to compression of the esophagus by the aberrant right subclavian artery (ARSA). It occurs due to embryologic anomalies of the brachial arches, which are often unrecognized. Dysphagia is the prime presenting symptom in the majority, in addition to the other tracheoesophageal symptoms. Dysphagia lusoria can be diagnosed using barium swallow and chest computed tomography (CT) scan. We report a case of a young male who presented with complaints of dysphagia.

## Introduction

Most cases of dysphagia lusoria are caused by posterior esophageal compression due to the aberrant right subclavian artery (ARSA). However, the presenting symptom in about 20-40% is dysphagia with other trachea-esophageal symptoms [1,2]. Here, we report a case of a young male who presented with complaints of late-onset non-progressive dysphagia, highlighting the significance of early diagnosis and management.

## Case presentation

**Figure 1:**
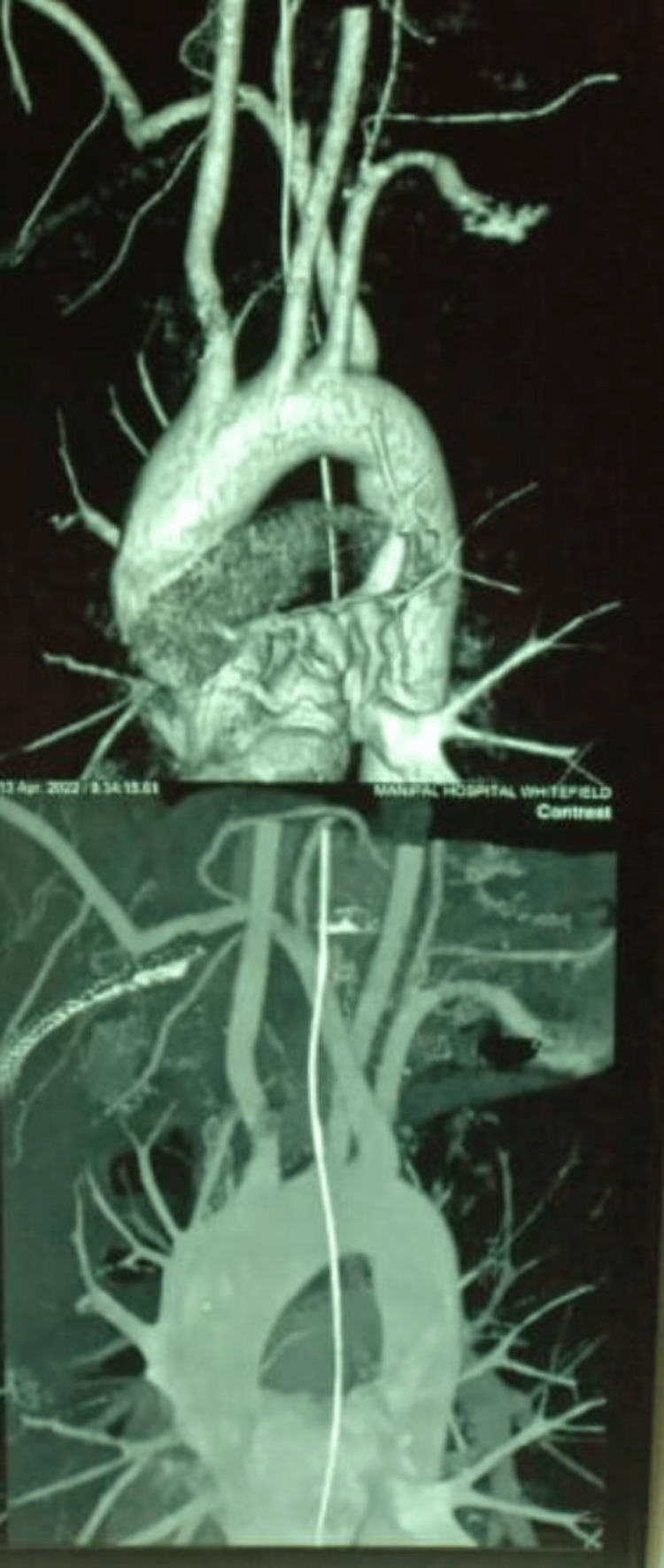
CT angiogram of the chest showing aberrant right subclavian artery

A 37-year-old male patient with no significant past medical and surgical history presented with complaints of non-progressive difficulty in swallowing (solids more than liquids) over six months. The patient was initially evaluated for mechanical causes of dysphagia, and hence upper GI endoscopy was performed, with no positive findings. The patient was then directed to the ear, nose, and throat (ENT) department, where indirect laryngoscopy was performed with no positive findings. The patient was further evaluated with CT angiography of the chest, which revealed an aberrant right subclavian artery (Figure 1). Consequently, the patient was diagnosed with a rare anatomical cause of dysphagia and was referred to Vascular surgery for further evaluation and management.

**Figure 2:**
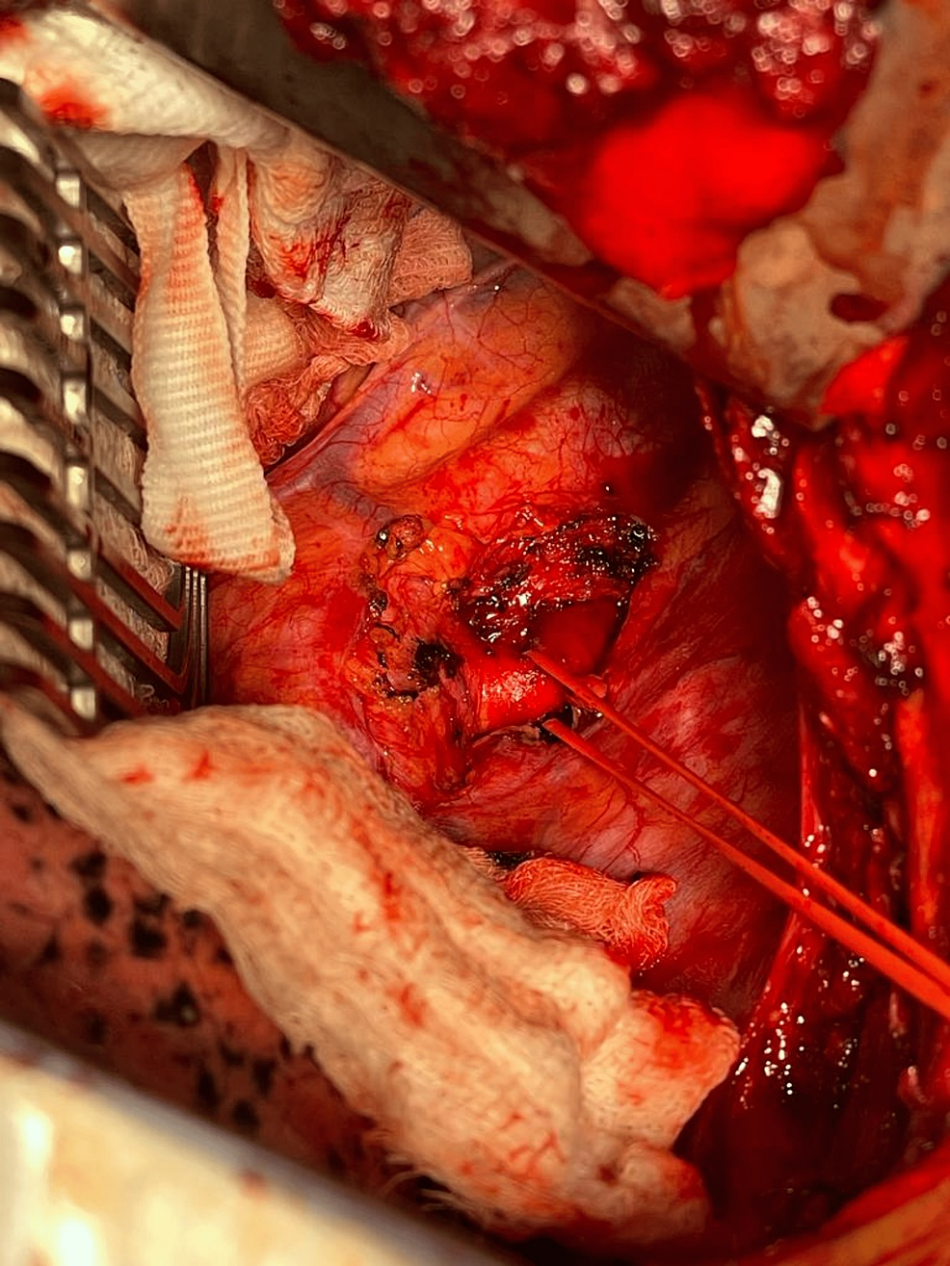
Right cervical approach with a vessel loop around the right common carotid artery

**Figure 3:**
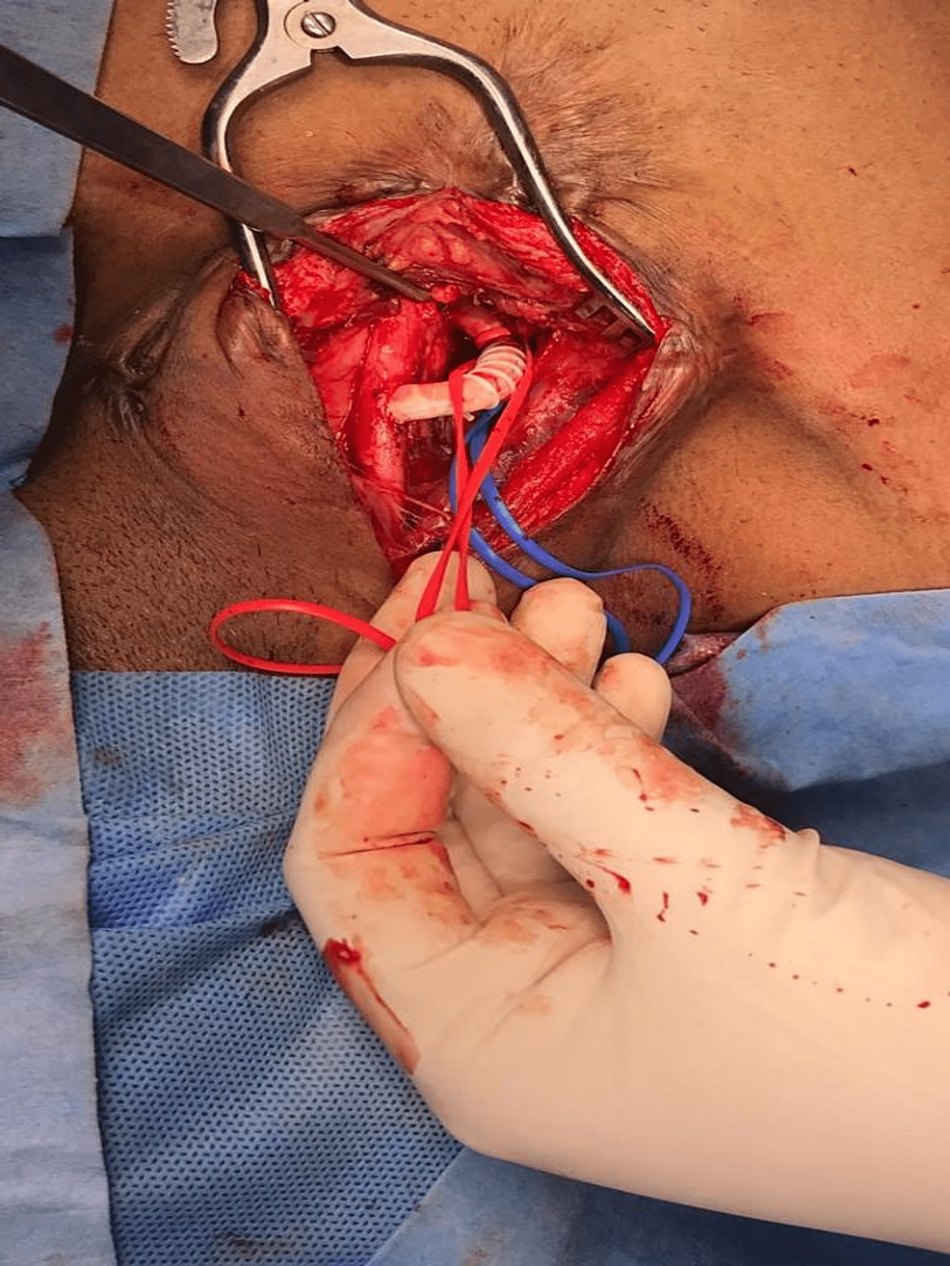
PTFE 6 mm graft showing the anastomosis between the right common carotid and the right subclavian artery PTFE - polytetrafluoroethylene

**Figure 4:**
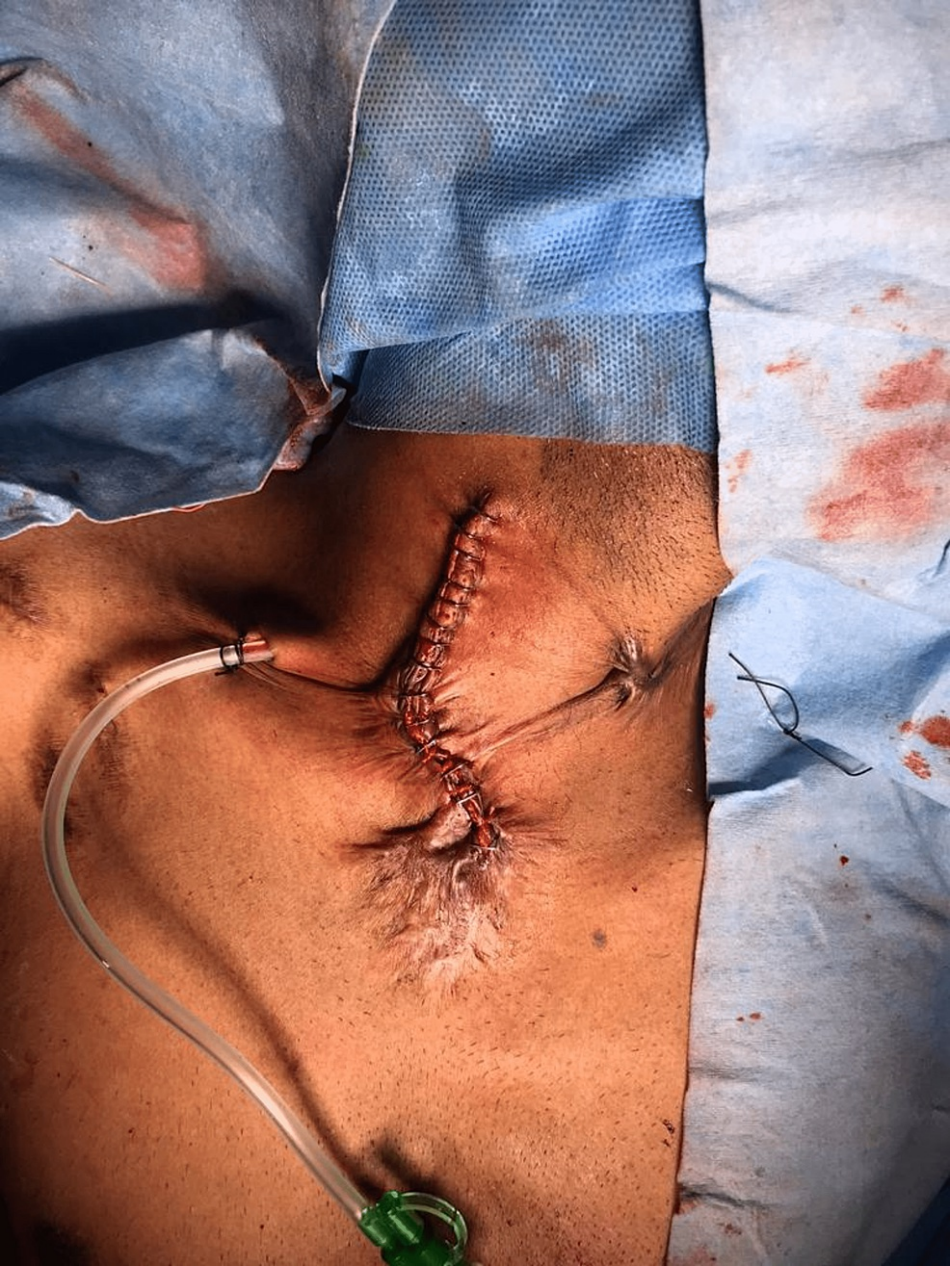
Closure of the right cervical incision post anastomosis with a drain in situ

**Figure 5:**
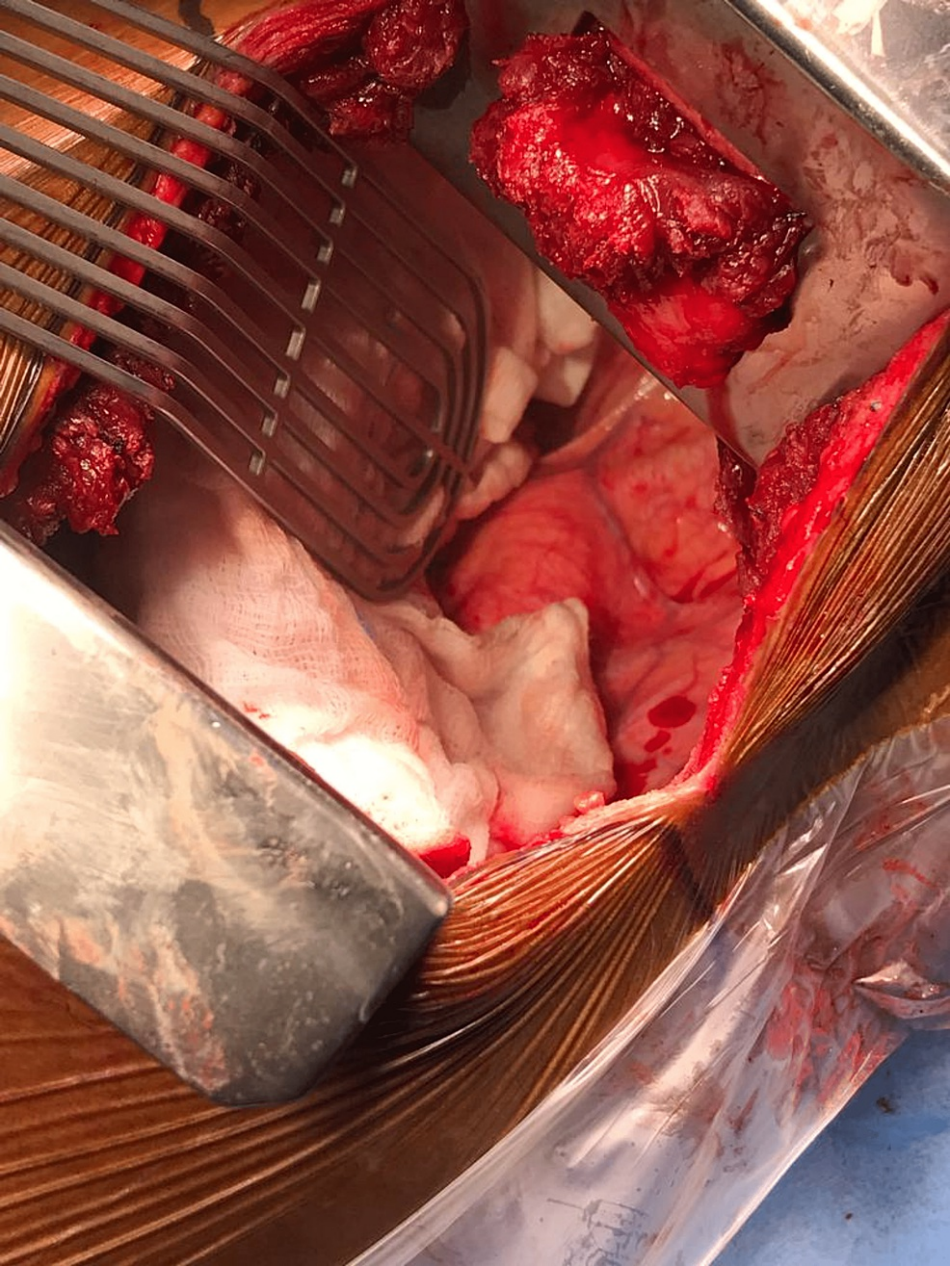
Left thoracotomy showing the origin of the aberrant subclavian artery

After that, the patient was further evaluated to strategize management, and a decision was made to manage the patient surgically. CT angiogram was studied in detail to locate the origin of the right vertebral artery. Since the artery was arising to the right side of the esophagus, a cervical approach was planned with a bypass graft between the right subclavian artery and the right common carotid artery. Through the right cervical approach, the right common carotid was exposed, and control was taken (Figure 2). Since the artery was deep, a short polytetrafluoroethylene (PTFE) 6 mm graft was used to connect the right common carotid artery to the right subclavian just proximal to the origin of the right vertebral artery (Figure 3). The subclavian artery was ligated proximally before the anastomosis, and the skin closed with drain-in situ (Figure 4). After that, a left thoracotomy was done, and the right subclavian artery was ligated at its origin from the aorta (Figure 5). A chest tube drain was placed. After closely monitoring the patient for a couple of days in the intensive care unit, the patient was shifted to the ward with stable hemodynamics.

On post-op day 3, the patient developed chylothorax, which was managed with octreotide in addition to daily drain output charting and a low-fat diet. The patient was discharged after a stable ward course of one week and was asked to follow up after a month. On the follow-up appointment, the patient stated that he was symptomatically much better and was swallowing solid food without discomfort. 

## Discussion

In the general population, ARSA is a vascular anomaly involving the aortic arch [2]. It is hypothesized that a persistent seventh intersegmental artery involutes the fourth vascular arch with the right dorsal aorta, thus forming ARSA [3,4]. As per published literature, the prevalence of the aberrant subclavian artery is around 0.7%. Predominantly, the aberrant artery courses posterior to the esophagus, but in a small percentage, it may run anterior to the esophagus [5].

The majority of dysphagia lusoria is due to the right subclavian artery originating from the left-sided aortic arch. Although infrequent, a similar abnormality can occur due to a left aberrant subclavian artery arising from a right-sided aortic arch [6,7]. This anomaly results from a patent right dorsal artery with abnormal regression of the left fourth arch or left dorsal aorta. When the abnormality is associated with left-sided ductus arteriosus, a complete vascular ring is formed, thus causing symptoms. Hence, dysphagia is the prime symptom in 30-40% of patients with vascular anomalies [3].

Patients with dysphagia lusoria are managed based on the symptom severity and the patient's propensity to support their weight and nourishment. Diagnostic tests like barium swallow studies have been used to screen dysphagia lusoria. Surgical intervention is planned using CT of the chest or magnetic resonance imaging as they help define the vascular lesion. However, manometric studies are not helpful in diagnosis. Various expositions, such as increased stiffness of the esophagus and the obstructing artery due to aging and motility disorders, cause late-onset dysphagia. In addition, Kommerell's diverticulum or aortic elongation with increased traction on the obstructing artery is another theory that exists [2,8].

Surgical intervention is justified for those with severe symptoms, not manageable by swallowing techniques and dietary adjustments. The first surgical management of this condition was reported by Gross in a four-month infant via left thoracotomy, describing the ligation of ARSA at its origin [9]. Dilation of the esophageal stricture using endoscopy is a safe alternative for patients reluctant to surgery. It provides symptomatic relief for a relatively long time and can be safely repeated multiple times upon recurrence [10].

## Conclusions

Dysphagia is a common symptom in daily clinical practice, and the possibility of ARSA as one of its causes is often missed. Early evaluation in patients who present with chest pain and dysphagia is crucial for diagnosis and favorable management. Despite the rare occurrence, it is prudent that the clinicians be aware of this atypical congenital vascular abnormality. The significance of early imaging, including CT angiography of the chest, is highlighted in this case to identify the origin of the aberrant subclavian artery causing esophageal compression.
